# Gut Microbiota Correlates With Clinical Responsiveness to Erythropoietin in Hemodialysis Patients With Anemia

**DOI:** 10.3389/fcimb.2022.919352

**Published:** 2022-07-22

**Authors:** Yifan Zhu, Yuyan Tang, Haidong He, Ping Hu, Weiqian Sun, Meiping Jin, Lishun Wang, Xudong Xu

**Affiliations:** ^1^ Department of Nephrology, Minhang Hospital, Fudan University, Shanghai, China; ^2^ Center for Traditional Chinese Medicine and Gut Microbiota, Institute of Fudan-Minhang Academic Health System, Minhang Hospital, Fudan University, Shanghai, China

**Keywords:** gut microbiota, ESRD (End Stage Renal Disease), Anemia, EPO hyporesponsiveness, hemodialysis

## Abstract

The main treatment for renal anemia in end-stage renal disease (ESRD) patients on hemodialysis is erythropoiesis (EPO). EPO hyporesponsiveness (EH) in dialysis patients is a common clinical problem, which is poorly understood. Recent searches reported that gut microbiota was closely related to the occurrence and development of ESRD. This study aims to explore the changes in gut microbiota between ESRD patients with different responsiveness to EPO treatment. We compared the gut microbiota from 44 poor-response (PR) and 48 good-response (GR) hemodialysis patients treated with EPO using 16S rDNA sequencing analysis. The results showed that PR patients displayed a characteristic composition of the gut microbiome that clearly differed from that of GR patients. Nine genera (*Neisseria*, *Streptococcus*, *Porphyromonas*, *Fusobacterium*, *Prevotella_7*, *Rothia*, *Leptotrichia*, *Prevotella*, *Actinomyces*) we identified by Lasso regression and ROC curves could excellently predict EH. In contrast, five genera (*Faecalibacterium*, *Citrobacter*, *Bifidobacterium*, *Escherichia–Shigella*, *Bacteroides*) identified by the same means presented a protective effect against EH. Analyzing the correlation between these biomarkers and clinical indicators, we found that gut microbiota may affect response to EPO through nutritional status and parathyroid function. These findings suggest that gut microbiota is altered in hemodialysis patients with EH, giving new clues to the pathogenesis of renal anemia.

## Introduction

Anemia is a common complication and a risk factor for the progression of chronic kidney disease (CKD) to end-stage renal disease (ESRD) (Webster et al., 2017). The degree of anemia in CKD patients tends to be parallel with decreased kidney function and more than 90% of ESRD patients have been diagnosed with anemia (Li et al., 2016). Causes of anemia in CKD are complex, but a central feature is the deficit of erythropoietin (EPO) produced by the kidney during ESRD. Impaired erythropoiesis also contributes to anemia due to poor response to EPO with reduced proliferative activity of erythroid precursors in bone marrow and erythrophagocytosis (Shih et al., 2018). The erythropoiesis-stimulating agents (ESA) and iron supplementation have been the common therapy for anemic patients with CKD. However, as many as 10% of patients with renal disease receiving ESA experience EPO hyporesponsiveness (EH) (Macdougall and Cooper, 2002), which has been associated with an increased risk of cardiovascular events and mortality rates (Solomon et al., 2010). Many factors had shown to be associated with EH such as iron deficiency, inflammation response, neocytolysis, and underdialysis (Marcelli et al., 2016). However, there is no definitive treatment for EH. Increasing the amount of EPO or iron therapy can bring side effects to patients: a high dose of intravenous iron increases mortality (Bailie et al., 2015); ESA with high doses increases the risks of stroke, vascular access thrombosis, and death (Besarab et al., 1998; Singh et al., 2006; Pfeffer et al., 2009). Some new drugs, such as hypoxic-inducible factor stabilizers, need more clinical trials to verify their safety. Therefore, other potential pathogenesis and treatment of EH and renal anemia need to be further studied to solve this clinical problem better.

The gut microbiota has been found to play crucial roles in many chronic inflammatory diseases, including obesity, type 2 diabetes, insulin resistance, atherosclerosis, and non-alcoholic fatty liver disease (Qin et al., 2012; Ridaura et al., 2013; Kazankov et al., 2019). Increasing evidence indicates an important role of the gut microbiota in the development of CKD. Compared to healthy persons, patients with CKD presented a significant reduction in the richness and structure of their gut microbiota (Li F et al., 2019). The gut microbiota of rats with 5/6 nephrectomy, as well as patients with ESRD on hemodialysis, exhibited differently from that of healthy controls (Vaziri et al., 2013). P-Cresyl sulfate and indoxyl sulfate, the most extensively studied uremic toxins derived from gut microbiota, have been reported to contribute to the decline of renal function (Ramezani and Raj, 2014).

Based on these findings, we hypothesized that gut microbiota in ESRD patients might be associated with EH and designed a pilot study to verify.

## Methods

### Study Cohort and Study Design

Two independent cross-sectional analyses were performed, namely, exploration and validation cohorts. This study was approved by our local ethics committee (ethics approval number 2021-056-01K). Thirty-one hemodialysis patients with poor responsiveness (PR) to ESAs and 34 hemodialysis patients with good response (GR) were consecutively enrolled as exploration cohorts from the hemodialysis center of the Minhang Hospital affiliated to Fudan University. Classification of the patients into poor or good responders was partly referred to the KDIGO Clinical Practice Guideline (Kliger et al., 2013). The inclusion criteria are as follows: All study subjects maintained regular dialysis for more than 6 months and were treated with the standard treatment of more than 10,000 units of intravenous ESAs twice a week for at least 3 months; patients with average hemoglobin (g/dl) of more than 110 in the last 3 months were enrolled in the GR group; patients with average hemoglobin of 110 or less in the last 3 months were enrolled in the PR group. Moreover, hemoglobin in both groups fluctuated by no more than 10%. Then, to validate the potential prediction effect for EH, an independent cohort composed of 16 hemodialysis patients with PR and 10 hemodialysis patients with GR were enrolled as a validation cohort from the same hemodialysis center. All subjects are of Shanghai Han nationality, and their geographic area and eating habits are similar. Moreover, all subjects maintained the recommended dietary habits of dialysis patients: appropriate amount of high-quality protein, sufficient calories, and a low-potassium diet (Ikizler et al., 2020). Since physical activities affect the gut microbiota, we selected patients who exercised three to five times a week as subjects.

**Figure 1:**
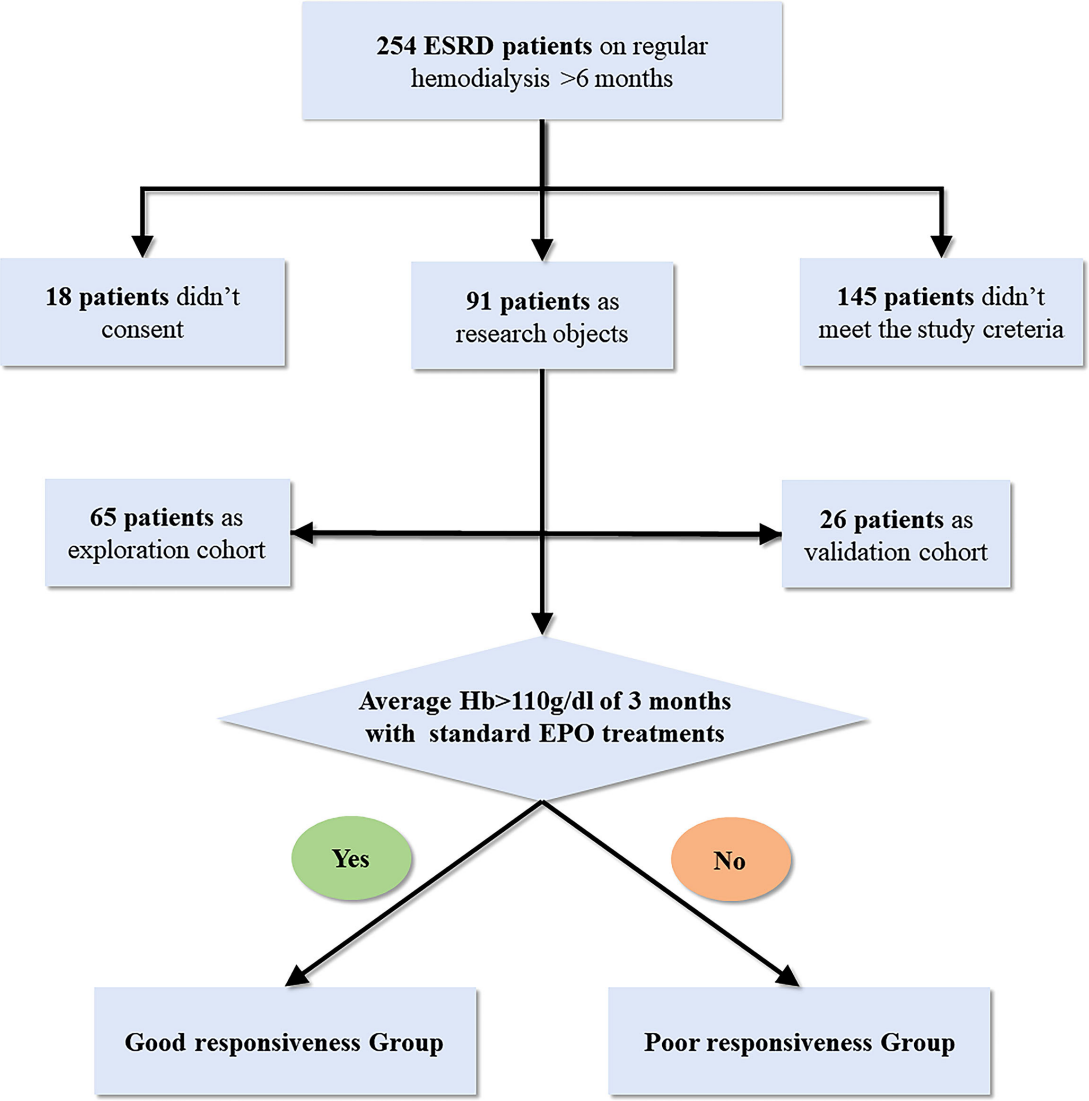
Flowchart of this study.

The exclusion criteria are as follows: (1) using oral antibiotics, probiotics, prebiotics, or synbiotics in the last 2 months; (2) history of tumor; (3) blood system disease; (4) gastroenteritis history in the past 3 months or other major digestive system diseases; (5) treatment of hypoxia-inducible factor–prolyl hydroxylase inhibitor; (6) serious CKD-associated complications; (7) definite etiology for EH, such as inflammation and EPO antibodies flowchart of this study was represented in **Figure 1**.

### Fecal Sample Collection and Laboratory Measurements

All participants were provided with sterilized 2-ml tubes containing bacterial cryopreservation fluid to collect fecal samples. Fecal samples were all freshly collected from patients in the morning of the second day after hemodialysis when the patients had an empty stomach and were delivered to Minhang Hospital affiliated to Fudan University not more than 4 h after collection. Fecal samples were quickly frozen at -80°C until DNA extraction after collection. Blood sampling was performed after fecal sample collection immediately. All serum parameters were measured using standard, provincially accredited laboratory techniques. Plasma levels of EPO and EPO-antibody were measured using commercially available ELISA kits based on the manufacturer’s instructions.

### DNA Extractions, PCR Amplification, and 16S rDNA Sequencing

DNA samples were subsequently aliquoted and extracted using the E.Z.N.A.^®^ Stool DNA Kit (D4015, Omega, Inc., USA). The reagent which was designed to uncover DNA from trace amounts of the sample has been shown to be effective for the preparation of DNA of most bacteria. Nuclear-free water was used for blank. The total DNA was eluted and stored at -80°C until measurement.

DNA amplification targeted the V3–V4 (Logue et al., 2016) regions using 341F (5′-CCTACGGGNGGCWGCAG-3′) and 805R (5′-GACTACHVGGGTATCTAATCC-3′) primers. The 5′ ends of the primers were tagged with specific barcodes per sample and sequencing universal primers. PCR amplification was performed in a total volume of 25 μl reaction mixture containing 25 ng of template DNA, 2.5 μl of each primer, 12.5 μl PCR Premix, and PCR-grade water to adjust the volume. The PCR products were confirmed with 2% agarose gel electrophoresis, purified by AMPure XT beads (Beckman Coulter Genomics, Danvers, MA, USA), and quantified by Qubit (Invitrogen, USA). The amplicon pools were prepared for sequencing on Agilent 2100 Bioanalyzer (Agilent, USA). The size and quantity of the amplicon library were assessed on the Library Quantification Kit for Illumina (Kapa Biosciences, Woburn, MA, USA) (https://support.illumina.com/documents/documentation/chemistry_documentation/16s/16s-metagenomic-library-prep-guide-15044223-b.pdf). The libraries were sequenced on NovaSeq 6000 PE250 platform.

### Processing of Sequencing Data

Samples were sequenced on an Illumina NovaSeq platform according to the manufacturer’s recommendations. Paired-end reads were assigned to samples based on their unique barcode and truncated by primer sequence and cutting off the barcode, then merged using FLASH (http://ccb.jhu.edu/software/FLASH/) (v1.2.8). Quality filtering on the raw reads was performed under specific filtering conditions to obtain the high-quality clean tags using fqtrim (http://ccb.jhu.edu/software/fqtrim/) (v 0.94). Chimeric sequences were filtered using Vsearch software (https://github.com/torognes/vsearch) (v2.3.4). After the dereplication of row reads using DADA2 (Callahan et al., 2016) (https://benjjneb.github.io/dada2/index.html), we acquired an amplicon sequence variant (ASV) table, a “higher resolution analogue of the OTU table.” According to the SILVA reference database (v 132) classifier (https://benjjneb.github.io/dada2/training.html), ASV abundance was normalized using the relative abundance of each sample. Alpha diversity is applied in analyzing the complexity of species diversity, including Chao1 and Goods coverage. Moreover, all these indices in our samples were calculated with QIIME2 (Rai et al., 2021). Beta diversity was calculated by QIIME2. The blast was used for sequence alignment, and the ASV sequences were annotated with the SILVA database for each representative sequence.

### Bioinformatics and Statistics Analysis

Gender distribution was analyzed by χ^2^ test, and other clinical indices were analyzed by Student’s t-test, using SPSS 20. Continuous variables are expressed as the mean ± standard deviation according to the normality of distribution. Statistical analysis of alpha diversity indexes between the two groups was performed with the Wilcoxon method. The differences between specific taxa of phylum and genus levels were determined using the Kruskal–Wallis test. The SparCC algorithm (Friedman and Alm, 2012), a correlation methodology developed specifically for microbial data, was computed using relative abundance profiles of the top 200 ASVs to estimate microbial associations between the two groups. Igraph in R (V3.6.1) was used for visualization of significant co-occurrence and co-excluding interactions (correlation coefficients <|0.5|, P < 0.05). LEfSe (linear discriminant analysis effect size) analysis was used to find the biomarkers with significant differences in abundance between GR and PR groups. LDA was utilized to examine the effect size of each differentially abundant trait, and a strict threshold of 4 was selected for logarithmic LDA scores. A Lasso (least absolute shrinkage and selection operator) (Mullah et al., 2021) regression model of genus-level biomarkers was applied to the data from the samples of GR and PR. Receiver operating characteristic curves (ROCs) were constructed, and the area under the curve (AUC) was calculated to determine the discriminatory ability of the Lasso regression model. Pearson correlation was used to analyze the relationship between the relative abundance of microbial markers and clinical indicators. Phylogenetic Investigation of Communities by Reconstruction of Unobserved States 2 (PICRUSt2) was used to predict microbial metagenomes and analyze the functional profiles of gut microbiota based on the 16S rDNA sequencing data. Briefly, we normalized ASVs and predicted the gene categories using the Kyoto Encyclopedia of Genes and Genomes (KEGG). P values <0.05 were considered statistically significant.

## Results

### Summary of Clinical Characteristics

Thirty-four patients with GR for ESAs and 31 patients with PR for ESAs were enrolled in this explorative cross-sectional study. Ten patients with GR and 16 patients with PR were subsequently included in the validation cohort. There were no statistically significant differences between the GR group and PR group in clinical features which included age, gender, BMI, residual renal function (RRF), EPO treatment age, EPO, and antibodies against EPO (EPO-Ab). Indicators associated with EH such as intact parathyroid hormone (iPTH), inflammatory index, and iron metabolism were not statistically different between the two groups. Notably, compared with the GR group, the iPTH, CRP, and transferrin saturation (TAST%) of the PR group all tend to increase, although they are all at normal levels. The summary of the characteristics of the patients is represented in **Table 1**.

**Table 1:** Summary of clinical characteristics.

	Exploration cohort			Validation cohort		
	GR	PR	P-value	GR	PR	P-value
**No. of patient**	34	31		10	16	
**Age, y**	59.1 ± 11.8	54.8 ± 14.6	0.191	59.7 ± 12.9	59.6 ± 12.3	0.979
**Sex (F/M)**	16/18	14/17	0.878	4/6	6/10	0.899
**EPO treatment age, y**	5.9 ± 2.1	6.0 ± 2.1	0.915	5.7 ± 2.6	5.4 ± 1.7	0.702
**Cause of kidney failure**						
**Diabetic nephropathy**	13	11		4	7	
**Glomerulonephritis**	15	16		5	8	
**others**	6	4		1	2	
**RRF (ml/min per 1.73 m²)**	5.4 ± 3.9	6.0 ± 4.5	0.589	6.3 ± 6.4	4.7 ± 3.2	0.981
**BMI**	22.5 ± 2.4	23.0 ± 3.2	0.549	21.3 ± 3.2	23.4 ± 3.3	0.115
**Hemoglobin (g/dl)**	117.5 ± 5.4	99.0 ± 9.0	<0.001	120.3 ± 2.9	102.6 ±5.4	<0.001
**Hematocrit (%)**	34.1 ± 3.9	33.9 ± 4.0	0.840	34.4 ± 3.4	34.6 ± 3.1	0.881
**CRP (mg/l)**	2.0 ± 2.3	3.1 ± 2.4	0.059	2.2 ± 1.9	2.4 ± 1.6	0.741
**EPO (ng/l)**	464.7 ± 150.5	468.5 ± 154.5	0.919	471.4 ± 182.7	481.9 ± 141.9	0.869
**EPO-Ab (ng/l)**	129.5 ± 39.8	146.9 ± 43.0	0.097	137.4 ± 25.6	154.5 ± 50.0	0.328
**Ferritin (ng/ml)**	394.5 ± 136.9	377.3 ± 95.2	0.561	371.0 ± 85.9	430.1 ± 101.0	0.137
**TSAT (%)**	32.5 ± 14.6	37.0 ± 22.3	0.328	25.9 ± 11.7	27.9 ± 13.1	0.704
**KT/V**	1.29 ± 0.2	1.2 ± 0.2	0.143	1.2 ± 0.1	1.2 ± 0.1	0.531
**Albumin g/l**	42.2 ± 1.6	41.7 ± 1.2	0.182	42.0 ± 1.6	41.1 ± 1.0	0.073
**iPTH** (**pg/mL)**	278.5 ± 248.6	381.0 ± 243.1	0.098	278.3 ± 171.2	309.5 ± 205.6	0.692
**Vitamin B12**	657.2 ± 213.3	564.8 ± 255.5	0.117	586.9 ± 171.2	519.9 ± 124.9	0.386
**Folate**	7.0 ± 3.1	6.1 ± 3.0	0.297	6.6 ± 2.5	6.4 ± 2.6	0.923

Gender distribution was analyzed by χ^2^ test, and other indices were analyzed by Student’s t-test. Data are expressed as the mean ± standard deviation according to the normality of distribution. The hemoglobin level is the 3-month average. GR, good response; PR, poor response. KT/V (clearance of urea multiplied by dialysis duration and normalized for urea distribution volume) was proposed as a parameter of dialysis adequacy.

### Different Microbial Structures Between GR and PR Groups

**Figure 2:**
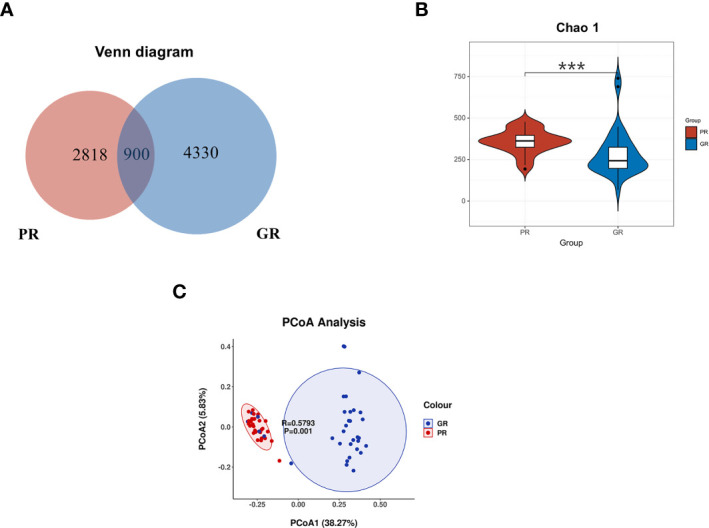
Comparison of the bacterial structure between the PR and GR groups. **(A)** Venn diagram presents the ASV numbers. **(B)** α diversity analysis of GR and PR. Chao1 index (P = 0.00018) revealed the ASV count. **(C)** PcoA analysis indicated that the microbiome samples were clustered in two groups. *p < 0.05; **p < 0.01; ***p < 0.001; ****p < 0.0001. PR, poor response; GR, good response.

In our present microbiome investigation, 16S rDNA gene PCR amplification of the V3–V4 regions was successful for all collected samples. A total of 3,502,655 sequences were obtained after quality filtering, merging, and chimera checking, with a median sequence count of 53,887 (range 32,957 to 66,973) per sample. The details (number) of the sequences that were paired match per sample in **Table S1**. According to the results of the ASV table, the Venn diagram visually presents the number of common and unique ASVs of each group (**Figure 2A**). The two groups shared 900 mutual ASVs. Among them, the GR group had 4330 ASVs, while the PR group had 2818 ASVs. To evaluate the differences in bacterial diversity between the two groups, sequences were aligned to estimate alpha diversity and beta diversity. Compared with the GR group, the PR group represented an increased α diversity estimated by Chao1 index. The Chao1 index (358.54 ± 181.65 vs. 287.48 ±136.9 P<0.001) mainly estimates the number of ASVs contained in the community (**Figure 2B**).

To examine the microbial community structure from patients with different responsiveness for ESAs, a non-parametric multivariate analysis of variance (Adonis) based on weighted UniFrac distances was performed between GR and PR groups. The calculated P-values (R^2^ = 0.3872, P = 0.001 for Adonis) further demonstrated significant differences in the microbial communities between the groups. The principal coordinate analysis (PCoA) revealed a separation of the two groups (**Figure 2C**). These results suggest that the diversity of gut microbiota could be strongly distinct in different EPO responsiveness of hemodialysis patients.

### Microbial Composition and Correlation Network Between GR and PR Groups

**Figure 3:**
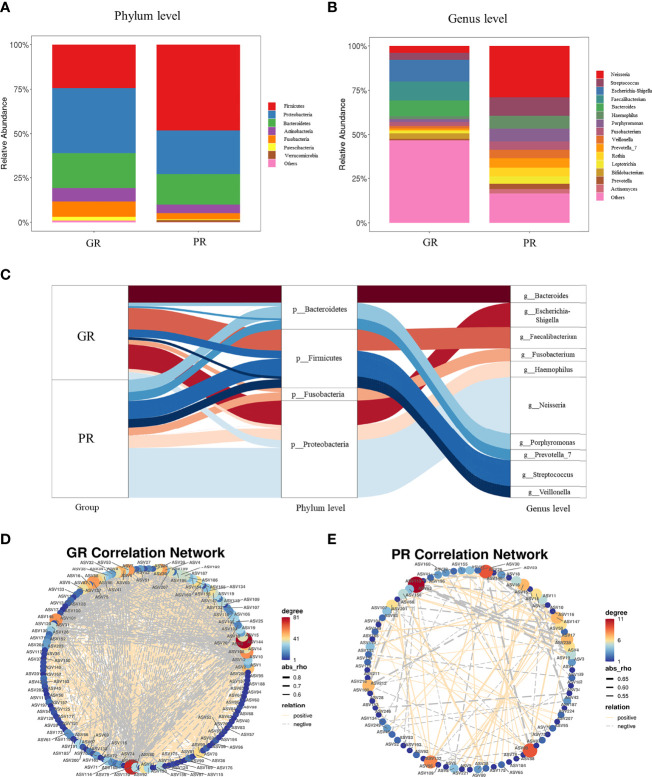
Comparison of the relative abundances at the phylum and genus levels. **(A, B)** The composition of bacteria at the phylum level **(A)** and at the genus level **(B)**. **(C)** Sankey plot visually showed the top relative abundance of bacteria at phylum and genus levels in both the GR and PR groups. **(D, E)** ASVs correlation network analysis. In total, SparCC correlation coefficients of |rho| >0.5 and P value <0.05 were used. The edge thickness indicates the strength of the correlation. The node size and color represent the number of other bacteria associated with the bacterium. PR, poor response; GR, good response.

Taxonomic analysis identified *Firmicute* (48.15% vs. 24.3% P < 0.001), *Proteobacteria* (24.82% vs. 36.83% P = 0.007), *Bacteroidetes* (17.04% vs. 19.56% P = 0.217), *Acinobacteria* (4.80% vs. 7.58% P < 0.001), and *Fusobacteria* (3.45% vs. 8.55% P < 0.001) as the most predominant taxa at the phylum level between GR and PR groups (**Figure 3A**). At the genus level, the most abundant taxa were *Neisseria* (3.85% vs. 27.30% P < 0.001), *Streptococcus* (3.91% vs. 9.84% P < 0.001), *Escherichia–Shigella* (12.25% vs. 0.02% P < 0.001), *Faecalibacterium* (10.68% vs. 0.02% P < 0.001), *Bacteroides* (8.86% vs. 0.01% P < 0.001), *Haemophilus* (1.66% vs. 6.86% P < 0.001), *Porphyromonas* (1.68% vs. 6.80% P < 0.001), *Fusobacterium* (2.28% vs. 4.54% P < 0.001), *Veillonella* (1.35% vs. 4.79% P < 0.001), *Prevotella_7* (1.02% vs. 4.92% P < 0.001), *Rothia* (4.46% vs. 0.54% P < 0.001), and *Bifidobacterium* (3.15% vs. 0.06% P < 0.001) between GR and PR groups (**Figure 3B**). The Sankey plot showed the relative abundance of the phylum level (middle) and genus level (right) of different samples (left), visually displaying the annotation information, corresponding relationship, and proportion of species at the two levels most concerned in the study of bacterial diversity (**Figure 3C**).

To further demonstrate the microbial co-occurrence and co-exclusion, we constructed the SparCC correlation of the top 200 ASVs at the genus level of GR and PR groups. As shown in **Figures 3D, E**, the bacterial genus correlations were distinct between GR and PR. Compared with genera in the PR, genera in the GR harbored a stronger correlation. The total number of edges was significantly greater in the GR network (n = 1262) than in the PR network (n = 111).

### Diagnostic Value of Genera as Biomarkers for EPO Hyporesponsiveness

**Figure 4:**
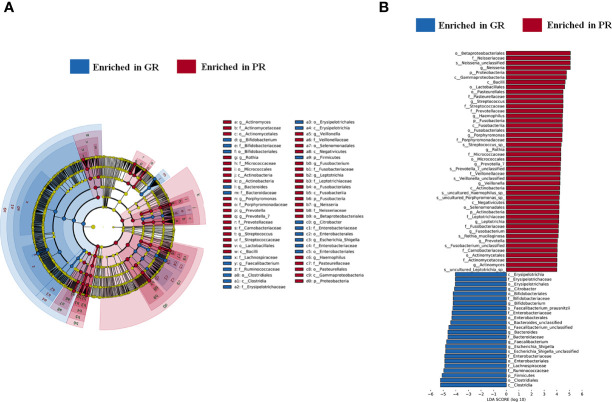
Marked differences in the abundance of gut microbiome at all taxonomic levels between GR and PR groups obtained by using the linear discriminant analysis (LDA) effect size (LEfSe) method; **(A)** The enriched taxa in GR and PR gut microbiome represented in the cladogram. The central point represents the root of the tree (Bacteria), and each ring represents the next lower taxonomic level (phylum to genus: p, phylum; c, class; o, order; f, family; g, genus). The diameter of each circle represents the relative abundance of the taxon. **(B)** Histogram of the LDA scores computed for differentially abundant taxa between GR and PR. The LDA score indicates the effect size and ranking of each differentially abundant taxon. P < 0.01, LDA >4. PR, poor response; GR, good response.

**Figure 5:**
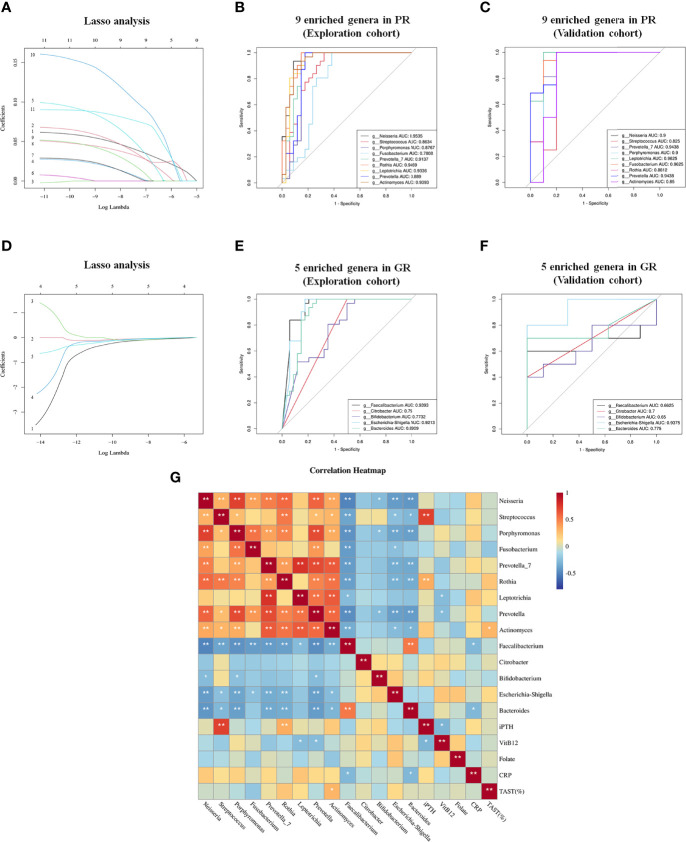
Lasso regression model and ROC to further identify biomarkers to distinguish GR from PR. **(A)** Lasso regression model of 11 genera filtered from LEfSe analysis; 1, *g:Neisseria* 2, *g: Streptococcus* 3, *g:Haemophilus* 4, *g:Porphyromonas* 5, *g:Fusobacterium* 6, *g:Veillonella* 7, *g:Prevotella_7* 8, *g:Rothia* 9, *g:Leptotrichia* 10, *g:Prevotella* 11, *g:Actinomyces*. **(B, C)** ROC curves of nine Lasso-selected genera in the exploration and validation cohorts with P < 0.05. **(D)** Lasso regression model of five genera enriched in GR filtered from LEfSe analysis. 1, *g:Faecalibacterium* 2, *g:Citrobacter* 3, *g:Bifidobacterium* 4, *g:Escherichia-Shigella* 5, *g:Bacteroides*. **(E, F)** ROC curve of five Lasso-selected genera in exploration and validation cohort with P < 0.05. PR, poor response; GR, good response. **(G)** Correlation analysis between biomarkers and potential indicators associated with EH. *p < 0.05; **p < 0.01.

To screen the diagnostic markers for the disease, we performed a taxonomic assignment of the sequences and analyzed each patient’s taxonomic profile, using the LEfSe algorithm representing significant differences between groups in the exploration cohort at all taxonomic levels (**Figures 4A, B**). According to stringent criteria for adjusted P-value < 0.01 and LDA score >4, 11 genera enriched in PR were selected as candidate biomarkers to predict the risk of EH in hemodialysis patients. Then, the Lasso regression model was performed on these 11 genera to predict EH (**Figure 5A**). Ten-fold cross-validation was applied to calculate the best lambda(λ), which leads to a minimum mean cross-validated error (**Figure S1A**). After verification, we screen out nine biomarkers of best λ: *Neisseria*, *Streptococcus*, *Porphyromonas*, *Fusobacterium*, *Prevotella_7*, *Rothia*, *Leptotrichia*, *Prevotella*, and *Actinomyces*.

To test whether these nine genera had diagnostic values for EH, the ROC curve was constructed. As shown in **Figure 5B**, all the biomarkers had a good diagnostic efficacy with the area under the curve (AUC) >0.75. Notably, *Neisseria* could distinguish PR from GR best, with an AUC of 0.9535 (95% CI, 90.2–100; P < 0.0001). Next, we verified the discriminatory power of the model using an independent validation cohort containing 16 hemodialysis patients with PR and 10 hemodialysis patients with GR. We observed a great diagnostic accuracy by using the same biomarkers from the exploration cohort (**Figure 5C**). Then, we identified the GR-enriched biomarkers according to the same method mentioned above (**Figure 5D**). Five genera (*Faecalibacterium*, *Citrobacter*, *Bifidobacterium*, *Escherichia–Shigella*, *Bacteroides*) enriched in the GR group had a protective effect on EPO responsiveness with the AUC >0.6 (**Figures 5E, F**). The evaluation of all biomarker combinations is summarized in **Supplemental Tables S2** and **S3**. The completed Lasso analysis and the ROC curves corresponding to each biomarker are clearly shown in **Figure S1**.

Since different biomarkers had different effects on EPO responsiveness, we constructed the Pearson correlation heatmap of these markers and clinical indictors associated with EPO responsiveness. As shown in **Figure 5G**, biomarkers (five genera) enriched in GR negatively correlated with biomarkers (nine genera) enriched in PR. Notably, *Streptococcus* and *Rothia* were positively correlated with iPTH (P < 0.01). *Leptotrichia* and *Prevotella* were negatively correlated with Vitamin B12. *Actinomyces* was positively correlated with TAST. *Faecalibacterium* and *Bacteroides* were negatively correlated with CRP.

### Functional Prediction of Gut Microbiota Between Two Groups

To provide a better understanding of the link between microbial dysbiosis and disease prognosis, we analyzed the KEGG pathways in bacterial populations with software PICRUSt2. In total, 35 level-2 KEGG pathways were found significantly different between GR and PR (**Table S4**). The PR group showed increased enrichment in pathways including replication and repair, translation, immune system, glycan biosynthesis and metabolism, nucleotide metabolism, metabolism of cofactors and vitamins, and energy metabolism. On the contrary, the GR group showed increased enrichment in pathways including carbohydrate metabolism, cell motility, cellular processes and signaling, environmental adaptation, enzyme families, immune system, membrane transport, signal transduction, transcription, xenobiotics biodegradation, and metabolism. Short-chain fatty acids (SCFAs) produced by gut microbiota fermentation are essential for maintaining gut mucosal homeostasis and closely related to human health. Therefore, we further predicted the main SCFA enzymes by the gut microbiota of the two groups using PICRUSt2 based on the KEGG ENZYME Database. As shown in **Figure S2**, most acetate, propionate, and butyrate synthesis-related enzymes presented a significant difference between GR and PR. Most butyrate synthesis-related enzymes were significantly enriched in GR. However, there was not a similar trend in acetate and propionate. These results demonstrated that GR may elevate butyrate *via* enriching the abundance of enzymes required for butyrate synthesis, which may be essential for its anti-anemia ability.

## Discussion

Recent studies have revealed that the gut microbiota plays an important role in the regulation of hematopoiesis. Compared with specific pathogen-free (SPF) mice, germ-free mice have smaller hematopoietic stem and progenitor cell (HSPC) populations (Ribeiro et al., 2016). Similarly, oral antibiotics have suppressive effects on hematopoiesis including anemia by depleting intestinal bacteria (Josefsdottir et al., 2017). There was a growing body of evidence to suggest that gut microbiota is involved in the development of CKD and ESRD as mentioned above, while such studies remain narrow in focus dealing only with CKD and ignoring the complications of CKD. However, poor prognosis of CKD is often due to uncontrolled complications such as cardiovascular events (Farrah et al., 2020), anemia (Vlagopoulos et al., 2005), and CKD-mineral and bone disorder (CKD-MBD) (Ketteler et al., 2017).

In this study, we applied 16S rDNA sequencing technology to characterize the gut microbiota among ESRD patients with different EPO responsiveness. A key result was that nine genera could predict EPO hyporesponsiveness (EH). We also found significant differences in the diversity, composition, and correlation of gut microbiota between ESRD patients with and without EH. The study subjects included the main primary diseases of renal failure: diabetic nephropathy, various chronic glomerulonephritis, polycystic kidney, hypertensive nephropathy, and uric acid nephropathy.

Comparison of the intergroup diversity of gut microbiota in the two groups, i.e., β diversity, could be completely separated, indicating that the two groups of microbiota belonged to two completely different populations. Although the total number of ASVs in the PR group was much lower than that in the GR group, the α diversity was slightly higher than that in the GR group. We believe that this phenomenon is due to the increase of harmful microbiota in PR, thereby increasing the α diversity. Although the α diversity of microbiota in the PR group was higher, the association between the microbiota was lower than that in the GR group according to the correlation analysis. This suggests that the microbiota of the PR group is more isolated and lacks interactions.

In a further study, we observed that the composition of bacteria at the level of genera was so different between the two groups, among which *Neisseria* were particularly prominent in the PR group, which may be pathogenic bacteria. A previous study had found that compared with healthy controls, ESRD patients exhibited a significantly higher relative abundance of *Neisseria* (Li Y et al., 2019). *Neisseria* has also been found to be involved in many diseases, such as hypothyroidism (Su et al., 2020), gastric cancer (Guo Y et al., 2020), and epilepsy (Safak et al., 2020). However, no studies have suggested a relationship between *Neisseria* and renal anemia or EPO response. Our results showed that *Neisseria* had the best predictive value for EH (AUC = 0.95) with a cutoff value of 6.15. *Streptococcus* has been reported to increase in various diseases, such as colorectal cancer (Gagniere et al., 2016), asthma (Ver et al., 2019), prediabetes (Allin et al., 2018), atherosclerotic cardiovascular disease (Jie et al., 2017), and some immune-mediated inflammatory disease (Forbes et al., 2018). We also observed that an increased relative abundance of *Streptococcus* could predict EH (AUC = 0.86). Accumulation of uremic toxins in ESRD will lead to sustained impairment of kidney function, inflammation, and endothelial dysfunction (Dou et al., 2004). Some uremic toxins, like indoxyl sulfate (IS) and P-cresyl derived from gut microbiota, exacerbate anemia in ESRD (Hamza et al., 2020). Previous studies have found that *Fusobacterium nucleatum* increased uremic toxin production and promoted renal disease development in a CKD rat model (Wang et al., 2020). We found a high abundance of *Fusobacterium* in the PR group, and it has a diagnostic value (AUC = 0.78). However, it is not clear whether *Fusobacterium* affects EPO reactivity by increasing metabolic toxins, which requires further study. Inflammation and pro-inflammatory cytokines had been reported to play an important role in EH patients (Casadevall, 1995; Macdougall and Cooper, 2002). Increased abundance of *Prevotella* was found to be associated with diseases featuring low-grade systemic inflammation, such as morbid obesity (Moreno-Indias et al., 2016), hypertension (Li et al., 2017), non-alcoholic fatty liver disease (Michail et al., 2015), and type 2 diabetes (Forslund et al., 2015). However, intestinal dysbiosis is associated with systemic inflammation in CKD patients (Mihai et al., 2018). Our results also found that *Prevotella* increased in the PR group and had a diagnostic value (AUC = 0.89).

In the good-response group, we observed an increase in one probiotic: *Bifidobacteria* (Messaoudi et al., 2011) with a predictive value (AUC = 0.77), suggesting that the addition of probiotics to the diet may be an adjuvant therapy for renal anemia and EH in ESRD. *Bacteroidetes* is an important part of commensal microbiota and plays a protective role in many diseases, such as virus infection (Stefan et al., 2020), colon cancer (Roberti et al., 2020), seizure (Olson et al., 2018), and gout (Guo et al., 2016). *Bacteroides* was also found to be decreased in the PR group, suggesting that EH may be due to the deficiency of normal commensal microbiota. Microbial networks may also reflect disease-specific microenvironments. The correlation network of the GR group was more complex than the PR group in both positive and negative correlations, suggesting that GR had a better microbial environment. Interestingly, predictive biomarkers enriched in the two groups presented mutual inhibition, suggesting that the growth of beneficial bacteria may be inhibited by the potentially pathogenic bacteria.

The cause of EPO hyporesponsiveness is unclear but may be related to hyperparathyroidism, inflammation, and nutritional status in CKD patients (Weir, 2021). Research indicates that there is an inverse correlation between PTH and Hb levels (Sliem et al., 2011), and elevated levels of PTH are significantly associated with EH in hemodialysis patients (Kalantar-Zadeh et al., 2009). However, the exact mechanism of hyperparathyroidism for EH remains unclear. In order to explore the mechanism of gut microbiota involved in EH, we analyzed the correlation of the identified microbial biomarkers with clinical indicators that may be related with EH. We found that *Streptococcus* and *Rothia* may be associated with increased iPTH to promote EH. Vitamin B12, folate, and iron are all important raw materials for hematopoiesis (Chaparro and Suchdev, 2019). The gut microbiome is a complex ecosystem that affects the development, immunological responses, and nutritional status of the host. Our results showed that *Leptotrichia* and *Prevotella*, which were enriched in PR, were negatively correlated with Vitamin B12. Moreover, *Faecalibacterium* and *Bacteroides*, which were enriched in GR were negatively correlated with CRP. Therefore, we speculated that gut microbiota may affect EPO reactivity through hyperparathyroidism, hematopoietic raw materials, and inflammation.

Recent advances showed that SCFAs produced by gut microbiota, especially acetate, propionate, and butyrate, have highlighted their effects on various systems at both cellular and molecular levels. Overmuch SCFAs or their deficiency may affect the pathogenesis of diseases, and adequate supplementation of SCFAs has protective effects on a variety of diseases (Tan et al., 2014). Many studies reported that host microbiota cross talk may contribute to the production and functional modulation of blood-cell lineages *via* producing SCFAs (Trompette et al., 2018; Guo H et al., 2020; Zhang et al., 2022). Our results showed that the butyrate synthesis-related enzymes were enriched in gut microbiota of GR. Sodium butyrate, a kind of histone deacetylase inhibitors, can reactivate fetal hemoglobin, stimulating the proliferation of red blood cells and considered for sickle cell anemia and beta-thalassemia therapy (Gabbianelli et al., 2000). Therefore, we speculated that better responsiveness to EPO or hemopoietic ability in GR may be due to better ability of synthesizing butyrate from gut microbiota. However, it is not clear whether SCFAs or butyrate can be used in renal anemia or EPO hyporesponsiveness.

However, this study has certain limitations. It is a single-center study, which lacks a large number of samples to verify. The samples are limited to hemodialysis patients, not involving abdominal dialysis and chronic kidney disease patients who have not yet entered the dialysis stage. In addition, 16s rDNA amplicon sequencing can only analyze taxonomically informative genetic markers for known and amplifying taxa. Therefore, it is difficult to analyze new or highly variable microorganisms. Although we detected species-level populations when analyzing differential markers, most of these populations were unclassified. Our results show that genus-level biomarkers are already excellent enough to predict EPO reactivity. Therefore, these species-level markers detected by 16s rDNA amplicon sequencing are not suitable as predictors.

## Conclusion

We found a strong correlation between gut microbiome and EH by observing the diversity and composition of gut microbiota. The biomarkers (*Neisseria*, *Streptococcus*, *Porphyromonas*, *Fusobacterium*, *Prevotella_7*, *Rothia*, *Leptotrichia*, *Prevotella*, *Actinomyces*) we identified have great effects in predicting EH in hemodialysis patients. Moreover, biomarkers (*Faecalibacterium*, *Citrobacter*, *Bifidobacterium*, *Escherichia–Shigella*, *Bacteroides*) presented a protective effect against EH may provide new insights into treatment. This study also found that these biomarkers may play a role by affecting nutritional status and parathyroid function. The findings in this study provide a new research direction of the mechanism and treatment of renal anemia: the intervention of gut microbiota is expected to be a novel therapy in CKD patients with clinical refractory anemia. Also, it can reduce the use of traditional medicine ESAs or iron agent, thereby reducing the adverse effects of these drugs, and improving the prognosis of patients with kidney failure.

## Data Availability Statement

The 16s rDNA sequencing data can be found in NCBI SRA repository, accession number PRJNA837984. Other data in this study can be found in the **Supplementary Material**.

## Ethics Statement

The studies involving human participants were reviewed and approved by Institutional ethics board of Fudan University Minhang Hospital. The patients/participants provided their written informed consent to participate in this study.

## Author Contributions

All authors confirmed they have contributed to the intellectual content of this paper and have met the following four requirements: (a) significant contributions to the conception and design, acquisition of data, or analysis and interpretation of data; (b) drafting or revising of the article for intellectual content; (c) final approval of the published article; and (d) agreement to be accountable for all aspects of the article, thus ensuring that questions related to the accuracy or integrity of any part of the article are appropriately investigated and resolved. YZ, LW, and XD conceived the study, designed the study protocol, and wrote the paper. YT, HH, and YZ performed the experiments. YT, HH, and WS analyzed the data. PH and MJ wrote the final version of the paper. All authors reviewed and approved the final version of the manuscript. YZ analyzed the data and prepared the manuscript, with editing and revision by all authors.

## Funding

National Natural Science Foundation of China (81774060); Shanghai Municipal Commission of Health and Family Planning (20184Y0040); Minhang District High-level Specialized Specialist Training Program (2020MZYS19); Fund of Minhang Health Commission (2020FM26).

## Conflict of Interest

The authors declare that the research was conducted in the absence of any commercial or financial relationships that could be construed as a potential conflict of interest.

## Publisher’s Note

All claims expressed in this article are solely those of the authors and do not necessarily represent those of their affiliated organizations, or those of the publisher, the editors and the reviewers. Any product that may be evaluated in this article, or claim that may be made by its manufacturer, is not guaranteed or endorsed by the publisher.
