# Supplementary material for: Gut Microbiota Correlates With Clinical Responsiveness to Erythropoietin in Hemodialysis Patients With Anemia
Source: Front Cell Infect Microbiol. 2022 Jul 22;12:919352. doi: 10.3389/fcimb.2022.919352 (PMC9355670; doi:10.3389/fcimb.2022.919352)
Supplement: Supplementary file 1 [file DataSheet_1.docx]

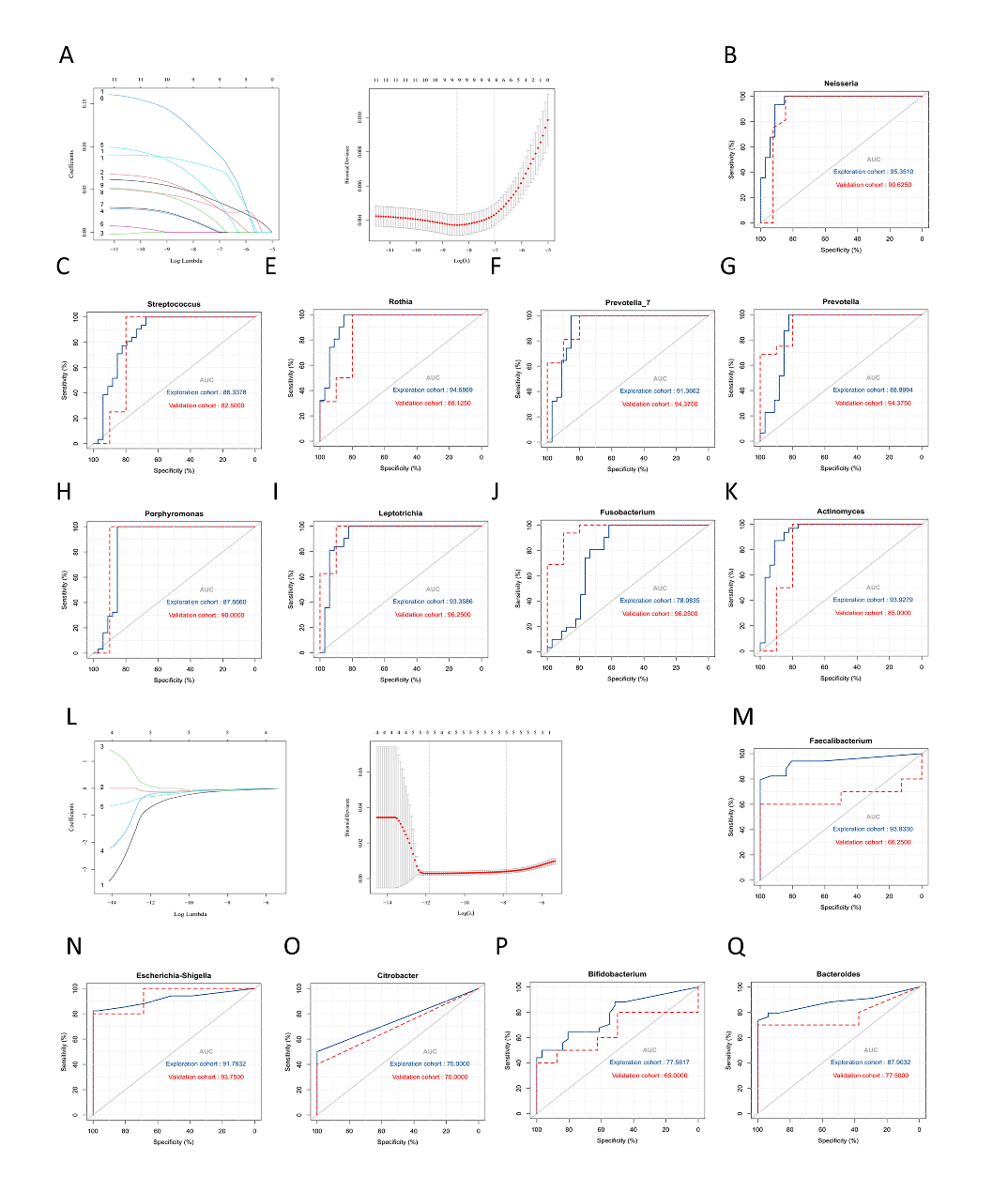


**Figure S1 Lasso analysis and ROC curves**

**(A)** Lasso regression model of 11 genera enriched in PR filtered from LEfSe analysis; Ten-fold cross-validation for the Lasso model.

1, *g__Neisseria* 2, *g__Streptococcus* 3, *g__Haemophilus*  4, *g__Porphyromonas*

5, *g__Fusobacterium*  6, *g__Veillonella* 7, *g__Prevotella_7*  8, *g__Rothia* 9, *g__Leptotrichia* 10, *g__Prevotella* 11, *g__Actinomyces*

**(B-K)** ROCs of 9 genera which distinguish PR from GR.

**(L)**Lasso regression model of 5 genera enriched in GR filtered from LEfSe analysis; Ten-fold cross-validation for the Lasso model.

1, *g__Faecalibacterium* 2, *g__Citrobacter* 3, *g__Bifidobacterium* 4, *g__Escherichia-Shigella* 5, *g__Bacteroides*

**(M-Q)** ROCs of 5 genera which distinguish GR from PR.


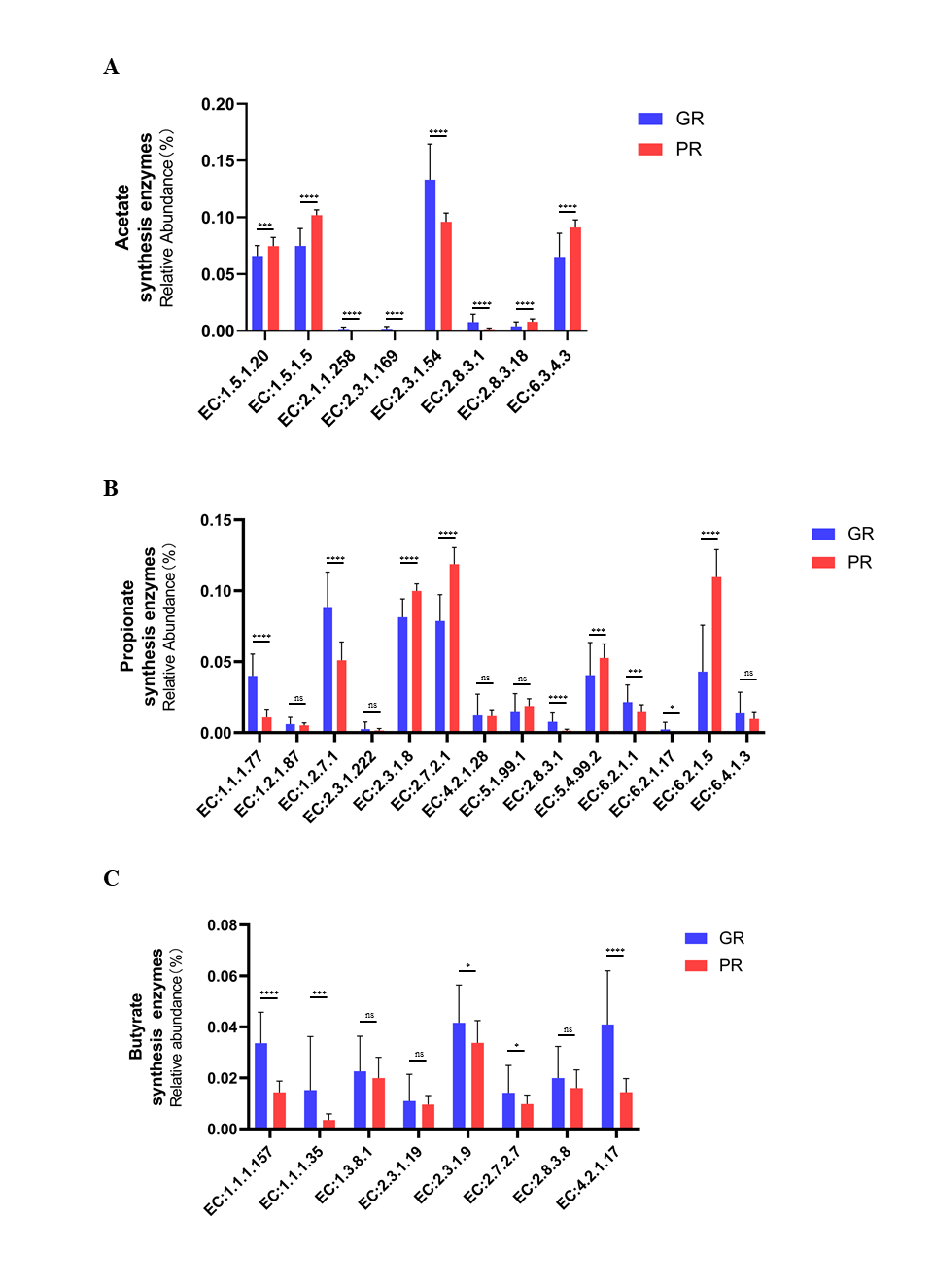


**Figure S2 SCFAs synthesis related enzymes from PICRUSt2**

(A) Acetate synthesis related enzymes from PICRUSt2.

(B) Propionate synthesis related enzymes from PICRUSt2.

(C) Butyrate synthesis related enzymes from PICRUSt2.

Ns, not significant; *P < 0.05;**P < 0.01;***P < 0.001;****P < 0.0001.

**Table S2 Evaluation of the 9 biomarkers combinations (in accordance with Figure 4C and 4D)**

|  | **Exploration cohort** | | | |  | |  | **Validation cohort** | | | |
| --- | --- | --- | --- | --- | --- | --- | --- | --- | --- | --- | --- |
|  | ***P-*value** | **95% CI** | **Sensitivity** | | | **Specificity** | | ***P-*value** | **95% CI** | **Sensitivity** | **Specificity** |
| **g__Neisseria** | ＜0.001 | 90.2-100 | 100 | 85.3  67.6  85.3  61.8  85.3  85.3  82.4  82.4  76.5 | | | | 0.007 | 62.6-99.5 | 100 | 69.2 |
| **g__Streptococcus** | ＜0.001 | 76.8-95.9 | 100 |  |  |  |  | 0.015 | 59-97.2 | 100 | 61.5 |
| **g__Porphyromonas** | ＜0.001 | 77.4-97.9 | 100 |  |  |  |  | 0.002 | 70.7-99.7 | 100 | 61.5 |
| **g__Fusobacterium** | ＜0.001 | 66-90.2 | 100 |  |  |  |  | 0.005 | 64.3-100 | 100 | 69.2 |
| **g__Prevotella_7** | ＜0.001 | 83.4-99.4 | 100 |  |  |  |  | 0.003 | 69.3-100 | 100 | 69.2 |
| **g__Rothia** | ＜0.001 | 89.1-100 | 100 |  |  |  |  | 0.026 | 54.7-96.8 | 100 | 61.5 |
| **g__Leptotrichia** | ＜0.001 | 86.4-100 | 100 |  |  |  |  | 0.015 | 58.5-97.7 | 100 | 69.2 |
| **g__Prevotella** | ＜0.001 | 79.8-98 | 100 |  |  |  |  | 0.003 | 68.2-100 | 100 | 61.5 |
| **g__Actinomyces** | ＜0.001 | 87.6-100 | 100 |  |  |  |  | 0.033 | 54.2-94.9 | 100 | 61.5 |

**Table S3 Evaluation of the 5 biomarkers combinations (in accordance with Figure 4E and 4F)**

|  | **Exploration cohort** | | | |  | |  | **Validation cohort** | | | |
| --- | --- | --- | --- | --- | --- | --- | --- | --- | --- | --- | --- |
|  | ***P-*value** | **95% CI** | **Sensitivity** | | | **Specificity** | | ***P-*value** | **95% CI** | **Sensitivity** | **Specificity** |
| **g__Faecalibacterium** | ＜0.001 | 87.9-99.9 | 100 | 79.4  50.0  50.0  82.4  73.5 | | | | 0.328 | 36.9-85.7 | 60 | 100 |
| **g__Citrobacter** | ＜0.001 | 62.9-87.1 | 100 |  |  |  |  | 0.15 | 44.9-88.4 | 40 | 100 |
| **g__Bifidobacterium** | ＜0.001 | 66.0-88.6 | 96.8 |  |  |  |  | 0.064 | 49.5-93.3 | 40 | 100 |
| **g__Escherichia-Shigella** | ＜0.001 | 84.9-99.4 | 100 |  |  |  |  | 0.0001 | 84.3-100 | 80 | 100 |
| **g__Prevotella_7** | ＜0.001 | 80.6-97.6 | 100 |  |  |  |  | 0.068 | 54.3-100 | 70 | 100 |

**Table S4 Gut bacterial functional profiles in terms of the KEGG pathways.**

| **KEGG pathways** | **Relative abundance(%)** | |  |  |
| --- | --- | --- | --- | --- |
|  | **GR** | **PR** | **P-value** | **sign** |
| Amino Acid Metabolism | 8.93±0.74 | 8.99±0.17 | 0.661 | ns |
| Biosynthesis of Other Secondary Metabolites | 0.84±0.12 | 0.75±0.04 | 0.000 | *** |
| Cancers | 0.1±0.01 | 0.11±0.01 | 0.456 | ns |
| Carbohydrate Metabolism | 10.06±0.76 | 8.8±0.27 | <0.001 | *** |
| Cardiovascular Diseases | 0±0 | 0±0 | <0.001 | *** |
| Cell Growth and Death | 0.53±0.1 | 0.62±0.02 | <0.001 | *** |
| Cell Motility | 2±0.66 | 1.08±0.15 | <0.001 | *** |
| Cellular Processes and Signaling | 4.64±0.44 | 4.12±0.18 | <0.001 | *** |
| Circulatory System | 0.01±0.02 | 0.05±0.01 | <0.001 | *** |
| Digestive System | 0.05±0.02 | 0.08±0.01 | <0.001 | *** |
| Endocrine System | 0.3±0.06 | 0.3±0.02 | 0.548 | ns |
| Energy Metabolism | 5.53±0.37 | 5.94±0.15 | <0.001 | *** |
| Environmental Adaptation | 0.16±0.03 | 0.12±0.01 | <0.001 | *** |
| Enzyme Families | 2.35±0.1 | 2.16±0.06 | <0.001 | *** |
| Excretory System | 0.02±0.01 | 0.03±0 | 0.010 | ** |
| Folding, Sorting and Degradation | 2.74±0.27 | 3.12±0.04 | <0.001 | *** |
| Genetic Information Processing | 3.02±0.27 | 3.27±0.07 | <0.001 | *** |
| Glycan Biosynthesis and Metabolism | 2.44±0.49 | 3.03±0.1 | <0.001 | *** |
| Immune System | 0.08±0.02 | 0.05±0.01 | <0.001 | *** |
| Immune System Diseases | 0.05±0.02 | 0.09±0.01 | <0.001 | *** |
| Infectious Diseases | 0.5±0.17 | 0.41±0.03 | 0.008 | ** |
| Lipid Metabolism | 2.77±0.13 | 2.84±0.09 | 0.014 | * |
| Membrane Transport | 12.08±2.06 | 10.72±0.39 | 0.001 | *** |
| Metabolic Diseases | 0.11±0.01 | 0.11±0 | 0.006 | ** |
| Metabolism | 2.29±0.37 | 2.04±0.07 | 0.001 | *** |
| Metabolism of Cofactors and Vitamins | 4.09±0.38 | 4.52±0.13 | <0.001 | *** |
| Metabolism of Other Amino Acids | 1.52±0.11 | 1.68±0.03 | <0.001 | *** |
| Metabolism of Terpenoids and Polyketides | 1.35±0.13 | 1.46±0.03 | <0.001 | *** |
| Nervous System | 0.1±0.03 | 0.07±0 | <0.001 | *** |
| Neurodegenerative Diseases | 0.14±0.08 | 0.33±0.05 | <0.001 | *** |
| Nucleotide Metabolism | 4.23±0.46 | 4.81±0.11 | <0.001 | *** |
| Poorly Characterized | 5.34±0.35 | 5.59±0.16 | 0.001 | *** |
| Replication and Repair | 9.39±1.07 | 10.67±0.12 | <0.001 | *** |
| Signal Transduction | 1.79±0.52 | 1.17±0.06 | <0.001 | *** |
| Signaling Molecules and Interaction | 0.19±0.04 | 0.23±0.02 | <0.001 | *** |
| Transcription | 2.86±0.43 | 2.16±0.06 | <0.001 | *** |
| Translation | 5.86±0.89 | 7.03±0.11 | <0.001 | *** |
| Transport and Catabolism | 0.26±0.1 | 0.28±0.01 | 0.311 | ns |
| Xenobiotics Biodegradation and Metabolism | 1.27±0.21 | 1.17±0.07 | 0.015 | * |

Notes: Data are expressed as the mean ± standard deviation according to the normality of distribution. GR, good response; PR, poor response.

Ns, not significant; *P < 0.05;**P < 0.01;***P < 0.001;****P < 0.0001.
